# Virtual screening for high affinity guests for synthetic supramolecular receptors†
†Electronic Supplementary Information (ESI) available: Details of guests **1–54**, and their binding constants in water; experimental information relating to the use of GOLD and XedeX; all calculated/measured binding constant data associated with Fig. 2, 3, 4 and 6. See DOI: 10.1039/c5sc00534e



**DOI:** 10.1039/c5sc00534e

**Published:** 2015-03-10

**Authors:** William Cullen, Simon Turega, Christopher A. Hunter, Michael D. Ward

**Affiliations:** a Department of Chemistry , University of Sheffield , Sheffield S3 7HF , UK . Email: m.d.ward@sheffield.ac.uk; b Biomedical Research Centre , Sheffield Hallam University , Sheffield S1 1WB , UK; c Department of Chemistry , University of Cambridge , Lensfield Road , Cambridge CB2 1EW , UK . Email: herchelsmith.orgchem@ch.cam.ac.uk

## Abstract

The protein/ligand docking software GOLD, which was originally developed for drug discovery, has been used in a virtual screen to identify small molecules that bind with extremely high affinities (*K* ≈ 10^7^ M^–1^) in the cavity of a cubic coordination cage in water. A scoring function was developed using known guests as a training set and modified by introducing an additional term to take account of loss of guest flexibility on binding. This scoring function was then used in GOLD to successfully identify 15 new guests and accurately predict the binding constants. This approach provides a powerful predictive tool for virtual screening of large compound libraries to identify new guests for synthetic hosts, thereby greatly simplifying and accelerating the process of identifying guests by removing the reliance on experimental trial-and-error.

## Abstract 2

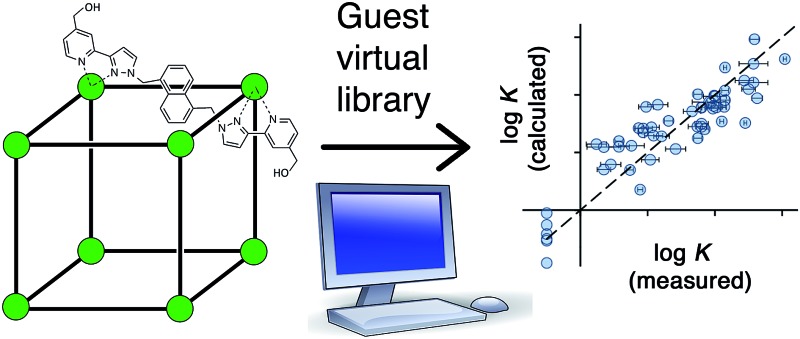
The protein/ligand docking programme ‘GOLD’ can be used to identify new strongly-binding guests for a synthetic coordination cage host.

## Introduction

Artificial container molecules, such as metal-based coordination cages and organic capsules, provide extensive opportunities for developing new types of functional behaviour based on binding of guest molecules in the central cavity.^1^ Since Cram first tamed cyclobutadiene inside an organic capsule,^2^ there have been numerous examples of how the reactivity of guest molecules can be modified by confinement in an environment that is quite different from that of the bulk solution,^3–5^ with seminal examples being Nitschke's stabilisation of P_4_ inside a cage cavity,^3*a*^ and the demonstration from Raymond and Bergman of enzyme-like catalysis in a cage cavity.^5^ Cages also have potential as drug delivery agents, with recent examples of binding,^6–8^ transport,^7^ and pH-controlled uptake and release of drug molecules.^8^ The future exploitation of container molecules will require an understanding of which guests will bind and how strongly. Systematic, quantitative approaches that put the contributions to guest binding in containers on a predictive footing are still in their infancy,^9,10^ so current studies rely on experimental screening of guests, which is inefficient and time-consuming.

Given the range of container molecules now in the literature for which applications based on guest binding are being sought, there is a clear need for improved *in silico* screening methods which would allow identification of complementary guests and prediction of association constants, providing leads for further study. Predictive tools for identifying compounds that bind to protein active sites are routinely used in drug discovery^11^ but have not been applied to synthetic systems. Given the potential for using such tools to understand the binding properties of container molecules and to provide predictability to guest binding, we set out to investigate the use of software developed for protein/small molecule interactions (GOLD) to predict binding affinities of guests in the cavity of a coordination cage.^12^

## Results and discussion

**Fig. 1:**
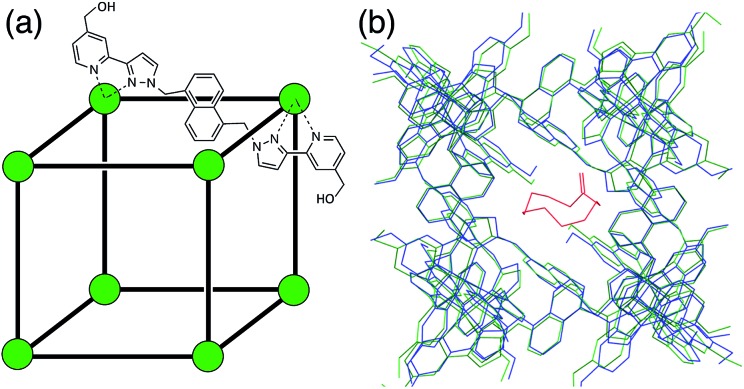
(a) Sketch of the cage showing the array of Co(ii) ions and the ligand structure; (b) overlay of the X-ray crystal structures of the cage containing only solvent molecules (blue), and containing cyclo-undecanone (green, with the guest in red). Solvent molecules and counterions are not shown for clarity.

The host cage that we used for this study is a [Co_8_L_12_](BF_4_)_16_ cage in which a Co(ii) ion occupies each vertex of a cube and a bridging ligand spans each of the edges (Fig. 1a).^10*c*^ The cage is functionalised with 24 hydroxyl groups on the external surface to make it water-soluble. It has a hydrophobic cavity with a volume of *ca.* 400 Å^3^, and there are portals in the faces of the cage, which allow guest access. The cage binds hydrophobic guests of the correct size and shape (*e.g.* aliphatic cyclic ketones, substituted adamantanes)^8,10a^ very effectively. The binding constant for cycloundecanone, that has a near-ideal volume for the cavity, is 1.2 × 10^6^ M^–1^.^10*a*^ This cage makes an ideal choice of host for our initial study. Not only do we have a large amount of empirical data on binding constants of various guests to use as a starting point (see below), but it is rigid with a geometrically well-defined cavity which simplifies calculation of host/guest complex structures, and it is soluble in water, the solvent for which GOLD was developed.

In the course of our studies on this cage,^8,10a,c^ we have used a combination of NMR titrations and a fluorescence displacement assay to measure binding constants for numerous guests in water. Our starting point for virtual screening is this set of 54 guests (**1–54**; see ESI, Fig. S1†), which provide the experimental data required for benchmarking a predictive model. For six of the 54 guests, binding interactions were not detected in water (*K* < 1 M^–1^). In order to include all of the systems in the study, the non-binding guests were therefore assigned a binding constant of 0.1 M^–1^, which is the lower limit for a solution phase interaction.^13^

In order to construct a target binding site for use in GOLD, we took the X-ray crystal structure of the cage^10*c*^ and removed the solvent molecules and counteranions. Fig. 1b shows an overlay of the X-ray crystal structures of the free cage and a complex where the cage contains a bound guest molecule.^8,10a^ With the exception of some of the side chains on the external surface of the cage, the structures show that the cage is rigid and does not change shape upon guest binding. X-ray crystal structures also show that the cage contains two specific binding sites for guest H-bond acceptors. For example, in the structurally-characterised complexes of the cage containing cycloundecanone and the cage containing adamantane carboxylic acid, the guest oxygen atoms are involved in several CH···O H-bonds with inwardly-directed C–H groups at these sites.^8,10^ We added a similarity acceptor constraint (see ESI†) in GOLD to force guest oxygen atoms to occupy these binding sites.

The application of docking software often requires modification of the default scoring function by training it against an experimental dataset to optimise the weightings of the individual contributions.^11^ We followed this approach, because the GOLD default scoring function (CHEMPLP)^14^ failed to predict the relative binding affinities of the training set of 54 guests. The CHEMPLP scoring function (eqn (1)) uses a piecewise linear potential to take into account steric complementarity between host and guest (ligand_clash), burial of a polar group in a non-polar environment (part_buried), hydrophobic interactions (non-polar), interactions of ligands with metal ions in the receptor (metal_coordination) and the torsional strain induced in the ligand on binding (ligand_torsion).^15^ There are also terms for hydrogen bonding interactions, which take into account the geometric dependence of these interactions (H-bond_donor and H-bond_acceptor).^16^

1 CHEMPLP score = *w*_lc_·*f*(ligand_clash) + *w*_pb_·*f*(part_buried) + *w*_np_·*f*(non-polar) + *w*_lt_·*f*(ligand_torsion) + *w*_mc_·*f*(metal_coordination) + *w*_hbd_·*f*(H-bond_donor) + *w*_hba_·*f*(H-bond_acceptor)

(where *w*_i_ are the weightings of each function, *f*).

**Fig. 2:**
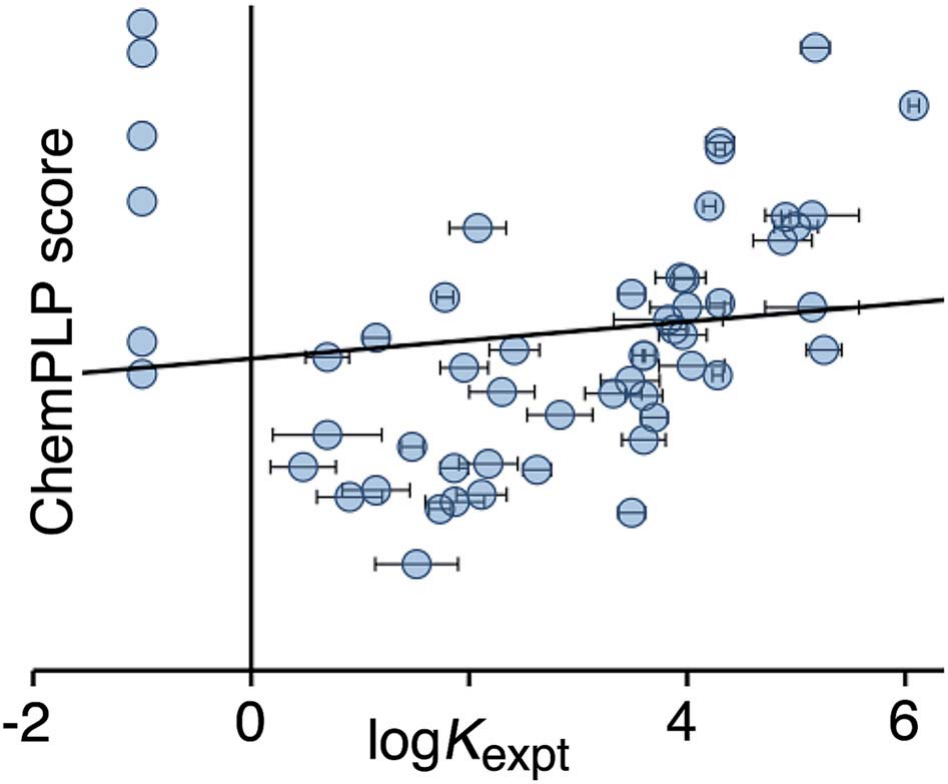
Comparison of experimental binding constants for the training set (*K*_expt_) with the CHEMPLP score calculated using eqn (1) (*r* ^2^ = 0.02). The solid line is the line of best fit. See ESI (Table S2†) for tabulated data.

Fig. 2 shows a comparison of the calculated CHEMPLP score and the experimentally measured binding constants for the training set. Although there is some correlation, there is very substantial scatter (*r* ^2^ = 0.02), and the non-binding guests perform particularly poorly.

In order to obtain a function that could be used to directly predict binding constants, we refined the weightings of the individual contributions in eqn (1) against the training set to optimise the scoring function. The result of this optimisation is eqn (2), which suggests that there are only four major contributions to binding in the cage: ligand_clash, ligand_torsion, non-polar, and part_buried (the numerical values calculated for these functions are given in ESI†). The importance of the non-polar term is consistent with our earlier empirical finding that guest binding in this cage in water is dominated by the hydrophobic effect.^10*a*^ The other terms in the CHEMPLP scoring function in eqn (1) relate to polar interactions, and the optimisation process gave all of these terms a weighting of zero, so they do not appear in eqn (2).

2 log *K*_calc_ = –3.83*f*(ligand_clash) + 0.12*f*(part_buried) – 0.08*f*(non-polar) – 2.71*f*(ligand_torsion)

**Fig. 3:**
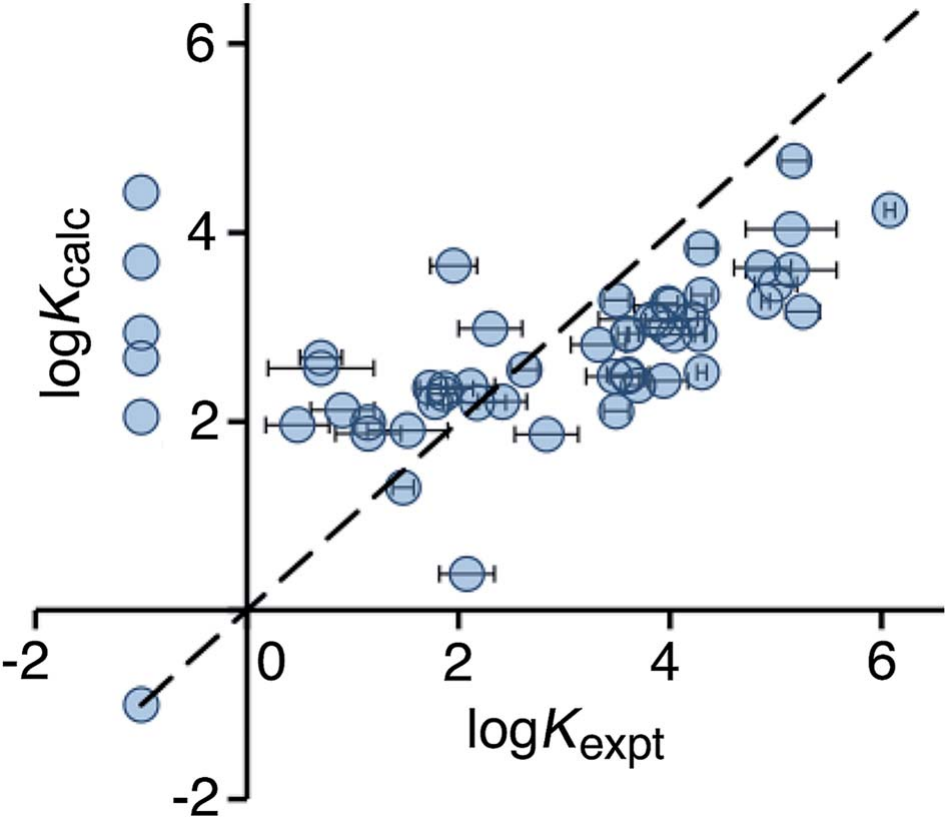
Comparison of experimental binding constants for the training set (*K*_expt_) with binding constants calculated using eqn (2) (*K*_calc_). The dotted line corresponds to *y* = *x* (RMSD = 1.66). See ESI (Table S3†) for tabulated data.

Use of eqn (2) significantly improves the correlation between calculation and experiment (*r* ^2^ = 0.21), and the result is illustrated in Fig. 3. For the high affinity guests, there is reasonable correlation between calculated and experimental binding constants. However, for five of the non-binding guests, the calculation still predicts erroneously high binding constants. These compounds are all open-chain molecules with high degrees of conformational flexibility. Based on their hydrophobic surface area, eqn (2) predicts binding constants for these guests that are comparable to those of more rigid guests, which have a similar hydrophobic surface area. For example, the linear (decan-2-one) and cyclic (cyclodecanone) C_10_ ketones are predicted by eqn (2) to bind with similar affinity. In practice, however, the cyclic ketone binds strongly (*K* = 1.5 × 10^5^ M^–1^) whereas the linear ketone shows no detectable binding in NMR titrations (*K* < 1 M^–1^).^10*a*^

In the GOLD docking process, a search of different guest conformations is performed, and it is possible to find a conformation of the open-chain ketone that fits as well into the cage as the cyclic ketone. The ligand_torsion term in eqn (2) describes the torsional strain, in other words the enthalpy penalty associated with putting a guest into a high energy conformation. However, the scoring function does not account for the entropy penalty of restricting degrees of freedom in an inherently flexible guest. To account for the loss of conformational mobility when flexible guests bind, we used the program XedeX to calculate the number of rotatable bonds in each guest (see ESI†).^17^ This number was used as an additional term, called ‘ligand_flexibility’, in the scoring function.

**Fig. 4:**
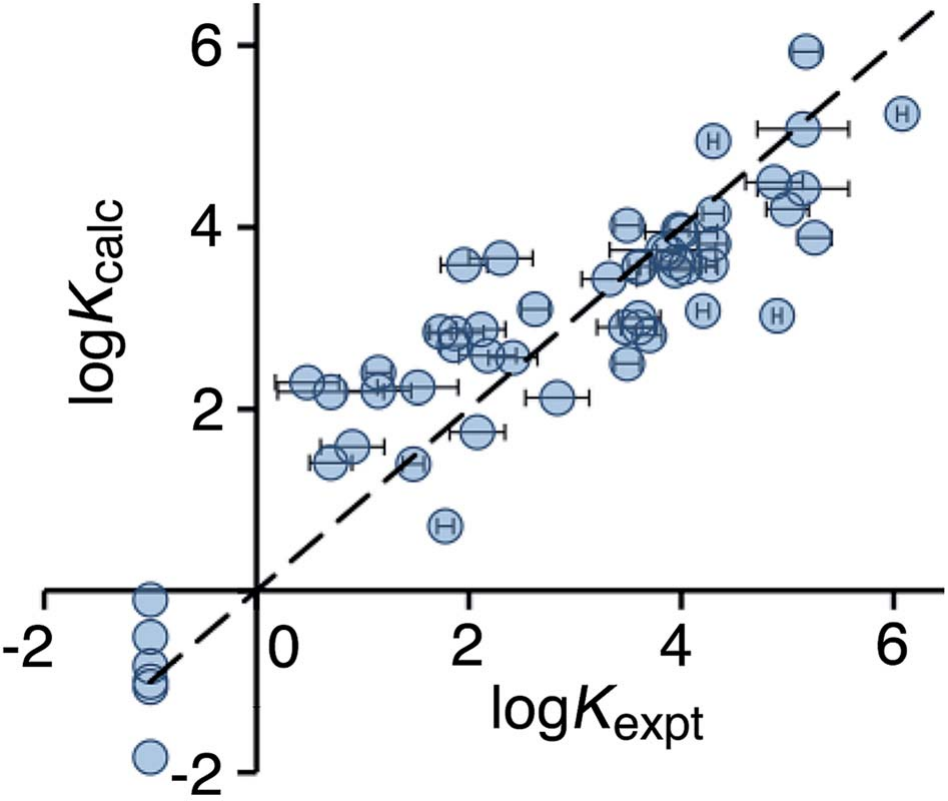
Comparison of experimental binding constants for the training set (*K*_expt_) with binding constants calculated using eqn (3) (*K*_calc_). The dotted line corresponds to *y* = *x* (RMSD = 0.79). See ESI (Table S4†) for tabulated data.

Optimisation of the new scoring function against the training set afforded eqn (3), which gives a much improved correlation between the calculated and experimental binding constants (Fig. 4). Specifically, the poor prediction of the binding properties of flexible guests has been corrected.

3 log *K*_calc_ = –4.48*f*(ligand_clash) + 0.20*f*(part_buried) –0.10*f*(non-polar) + 0.90*f*(ligand_torsion) –0.93*f*(ligand_flexibility)

**Fig. 5:**
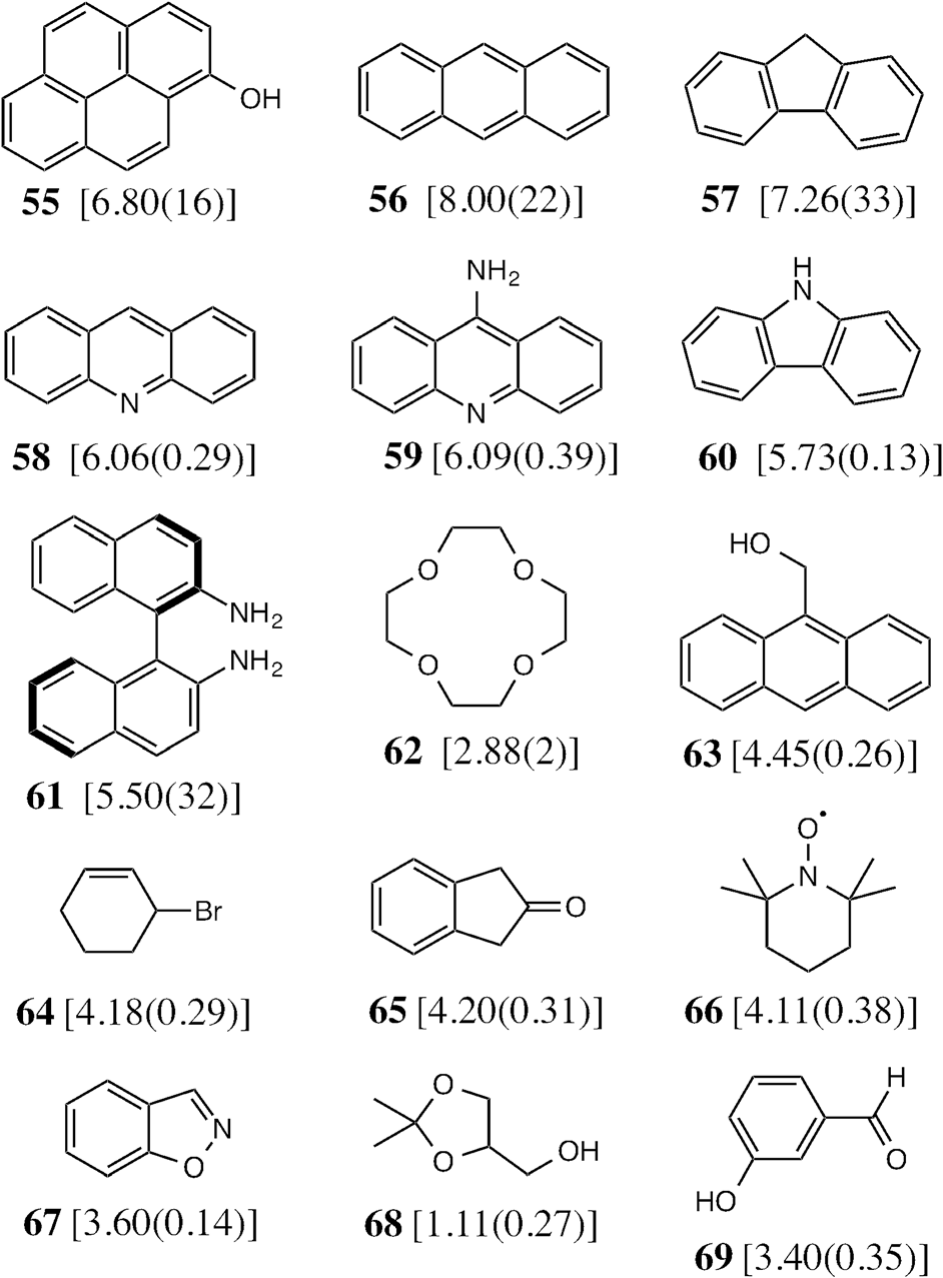
The 15 new guests identified by an in-house library screen of 3000 compounds using the scoring function in eqn (3). The experimentally measured log *K* values in water (with errors) are shown in square brackets.

**Fig. 6:**
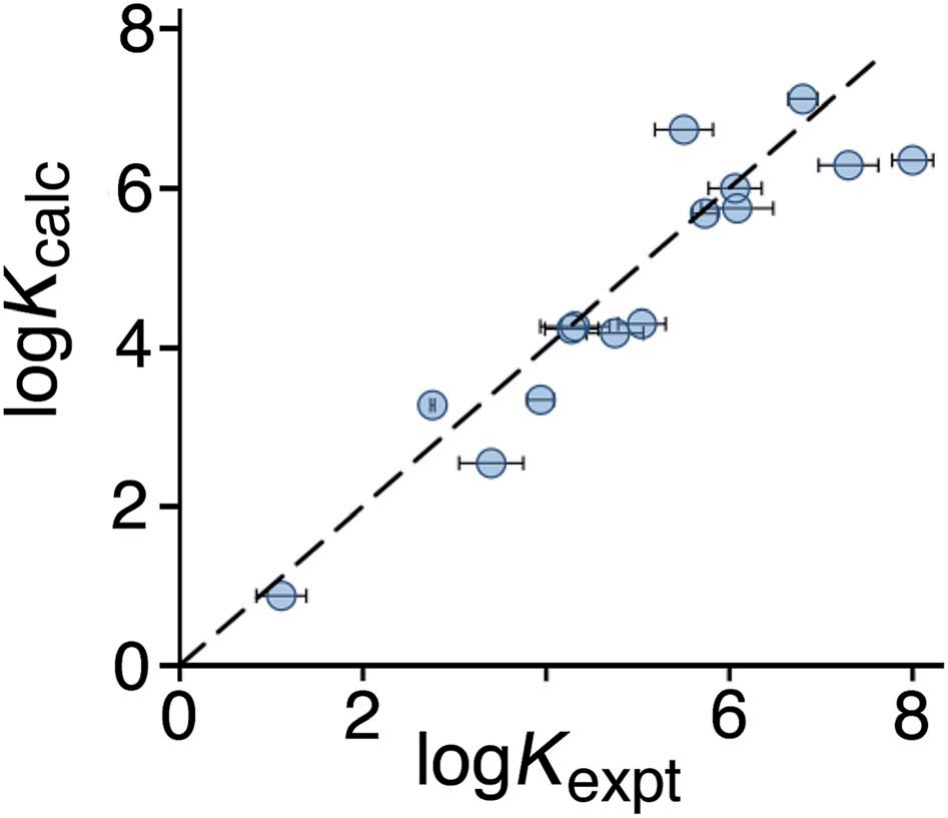
Comparison of experimental binding constants for the 15 new guests in Fig. 5 identified using GOLD (*K*_expt_) with binding constants calculated using eqn (3). The dotted line corresponds to *y* = *x* (RMSD = 0.79). See ESI (Table S5†) for tabulated data.

To test the predictive ability of eqn (3), we screened an in-house library of *ca.* 3000 compounds to identify potential new guests. From this screen, we selected 15 compounds (**55–69**, Fig. 5) that were predicted to bind with log *K* values in the range 0.9–7.1. Binding constants for these were measured using either NMR titrations or fluorescence displacement assays in water,^8,10^ and the results are included in Fig. 5 (the titration data fit well to a 1 : 1 binding isotherm in all cases). The correlation between predicted and measured binding constants for this set of 15 guests (Fig. 6) is very good and clearly shows the predictive value of GOLD for identifying new guests. The RMSD for the training set of 54 known guests (0.79) is identical to the RMSD for the new set of 15 guests. This is particularly encouraging, because the new guests include classes of compound that were not present in the original training set: several polycyclic aromatics, and compounds with no polar groups (**56** and **57**). Several of the new guests identified by GOLD in this single screen bind more strongly than our previous best guest (cycloundecanone, log *K* = 6.1)^10*a*^ which was the culmination of hundreds of experimental measurements. The new guests include classes of compound that we had not previously considered, and include several well-known fluorophores; a stable radical (TEMPO, **66**); and a crown ether (**62**) which is itself a host for small metal ions – all of which suggest interesting new avenues for exploration in the physical properties of supramolecular assemblies.

## Conclusion

In conclusion, we have demonstrated for the first time that docking software, developed for the analysis of protein/ligand interactions in drug discovery, can be used to identify new guests for a synthetic supramolecular receptor and accurately predict binding constants to within an order of magnitude. A training set of 54 guests was used to optimise a GOLD scoring function, which included a new term to account for the loss of conformational mobility when flexible guests bind. The scoring function is unique to this host, but the process of developing a scoring function is sufficiently straightforward that, given (i) a rigid host with a three-dimensional structure from crystallography or molecular modelling, and (ii) enough known guests to provide an initial training set, a scoring function specific to any synthetic receptor can be developed in the same way. The approach is not limited to water-soluble systems, and it should be possible to develop GOLD scoring functions for use in different solvents.

This methodology creates the possibility for guest binding in artificial molecular containers to be predictable and for new guests to be identified with confidence by virtual screening. The ability to predict host–guest interactions reliably will in turn open the door to a massive expansion of possible types of functional behaviour that can be developed with molecular containers and allow synthetic hosts to achieve their full potential.
