# Supplementary material for: Virtual screening for high affinity guests for synthetic supramolecular receptors
Source: Chem Sci. 2015 Mar 10;6(5):2790–4. doi: 10.1039/c5sc00534e (PMC5489031; doi:10.1039/c5sc00534e)
Supplement: Supplementary file 1 [file SC-006-C5SC00534E-s001.pdf]

Electronic Supplementary Material (ESI) for Chemical Science.
This journal is © The Royal Society of Chemistry 2015

Virtual Screening for High Affinity Guests for Synthetic Supramolecular Receptors

William Cullen,[a] Simon Turega,[a] Christopher A. Hunter* and Michael D. Ward*

Supporting Information

Experimental details associated with the calculations

Host ('Protein')

We used the previously-derived crystal structure of the cage (see main text) as the 'protein' by importing the coordinates into GOLD as a .mol2 file. Solvent molecules and anions were removed such that only the cage cation was considered.

Guests ('Ligands')

We first created the SMILES strings that describe the guest molecules by using ChemCell[1] (a Macro that enables Microsoft Excel to convert columns of chemical names and CAS Numbers into SMILES strings). We then used the program TORCH[2] to generate the 3D minimised structures for each of the guest molecules. These molecules were exported as a combined .mol2 file for use in GOLD, and as a combined .sdf file for use in XedeX.[3] The structures were visually checked to ensure they had been created successfully.

Running GOLD

The Host and Guest sets were then imported to GOLD as the 'Protein' and 'Ligands' respectively. The binding site was defined by using the cobalt atoms to locate the centre of the cavity; the scoring function was selected (in this case ChemPLP); and the .conf file was created ready to be run in GOLD. This was all done by following GOLD's built-in wizard.

A positioning constraint for the guest was added to locate H-bond acceptors such as carbonyl groups in one of the two H-bonding pockets in the corners of the cage cavity. This was achieved by including (as a .mol2 file) the two solvent molecules (MeOH in this case) that occupied these binding sites in the crystal structure. We added to the end of the GOLD .conf file the line:

"constraint similarity acceptor C:/location/solvent.mol2 10"

The .conf file was subsequently run through GOLD saving one solution per ligand, and the outputs were exported as a .csv file for use in Microsoft Excel.

Calculating the number of rotors (our 'ligand_flexibility term')

Using the .sdf file produced by TORCH for the set of guests, we used Babel[4] to separate the molecules into individual .pdb files which were subsequently run through XedeX (using an in-house Linux computer cluster) to calculate the number of independent rotors for each guest. A script was written to take the individual output files into a combined output .txt file. The number of rotors for each guest was copied from this file into Microsoft Excel and used as the 'ligand_flexibility' term along with the other terms output by GOLD (see Table 1, final column).

Generating the Scoring function

We used Microsoft Excel's 'solver' add-on to do a non-linear least-squares regression analysis, in which the weightings of the different contributions to the scoring function (see Table 1) were varied to minimise the sum of the squares of the errors between the calculated and experimental log*K* values.

Fig. S1: The initial 'training set' of guests (see later tables for measured binding constants). Of these the following binding constants in the host cage in water have been reported before:
1 – 7, 9, 10, 12: ref. 10c from main text
14 – 32, 36, 38: ref. 10a from main text
37, 39, 40, 41: ref. 8 from main text.
The remaining measurements are new in this work.

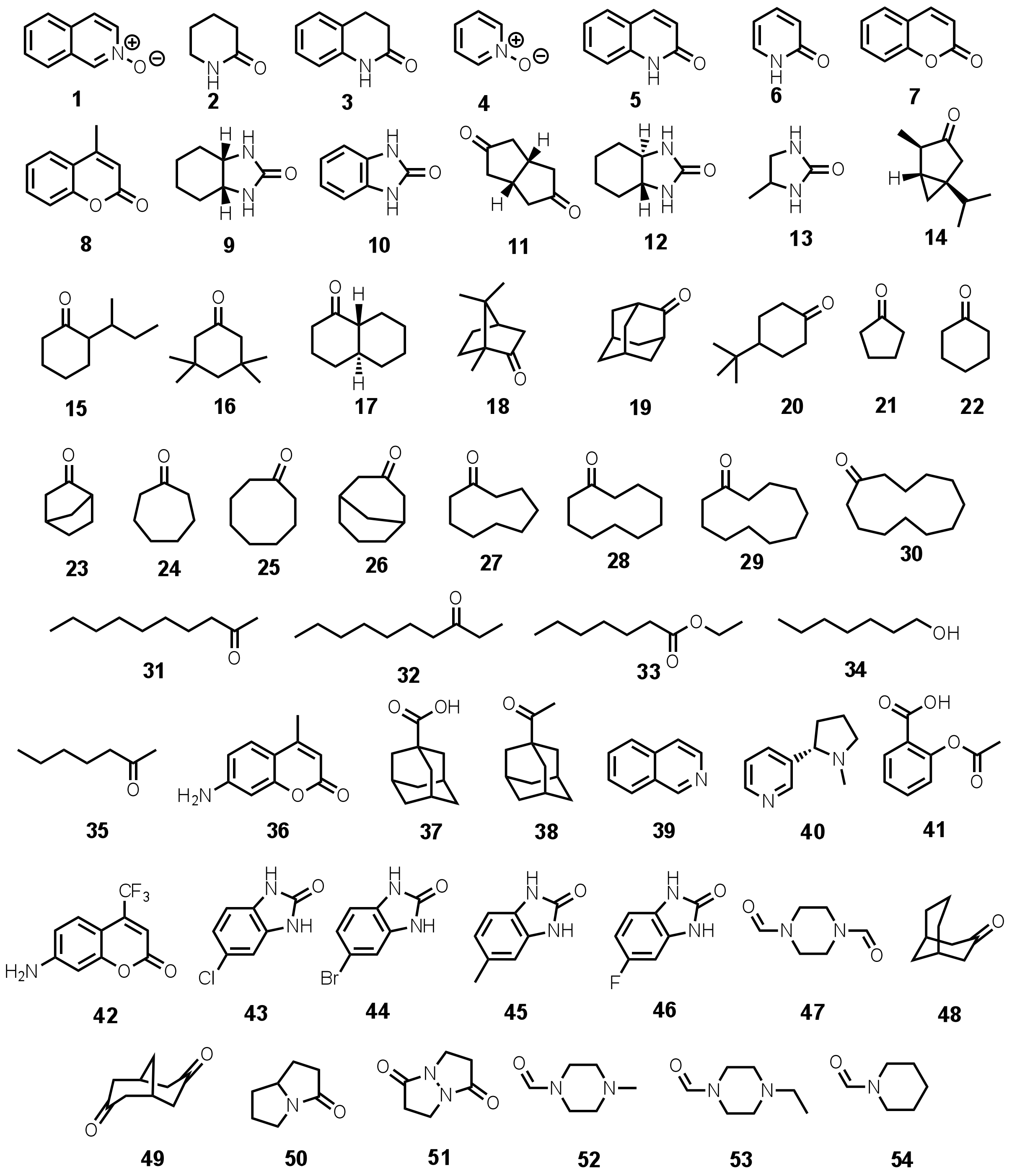

Table S1. Numerical values generated by GOLD for the individual terms that contribute to the scoring functions (main text, eq. 2 and 3).

Guest	*Ligand_clash*	*Ligand_torsion*	*Part_buried*	*Non-polar*	*Ligand_ flexibility*
1	0	0	-2.109	-43.3137	0
2	0	0	-3.0465	-27.4388	0
3	0	0	-2.4099	-41.2634	0
4	0	0	-2.26	-32.0514	0
5	0	0	-2.7351	-40.6438	0
6	0	0	-3.1134	-27.9508	0
7	0	0	-2.4121	-41.0365	0
8	0	0	-2.7123	-46.2622	0
9	0	0	-3.4782	-33.1938	0
10	0	0	-2.9428	-30.1587	0
11	0	0	-4.2726	-29.1526	0
12	0	0	-3.4382	-32.0023	0
13	0	0	-4.6213	-22.7748	0
14	0	0.6359	-0.5272	-52.666	1
15	0	0.2683	-1.9122	-49.4866	2
16	0	0	-1.2999	-46.3249	0
17	0	0	-2.2249	-43.0767	0
18	0	0	-1.7939	-41.3553	0
19	0	0	-1.7048	-38.256	0
20	0	0.3499	-2.0598	-44.3617	1
21	0	0	-1.9986	-27.2969	0
22	0	0	-2.2161	-32.0919	0
23	0	0	-2.1068	-32.1512	0
24	0	0	-1.9886	-34.0851	0
25	0	0	-2.0557	-37.4516	0
26	0	0	-2.2293	-39.0059	0
27	0	0	-2.0968	-38.8338	0
28	0	0	-2.0458	-47.1174	0
29	0	0	-1.708	-54.4098	0
30	0	0	-1.1725	-60.0173	0
31	0	0.2147	-2.2221	-55.531	7
32	0	0.0484	-2.0233	-58.793	7
33	0	0.6156	-3.0882	-50.0399	6
34	0	0.0352	-2.0632	-40.2044	6
35	0	0.0866	-2.1602	-38.7078	4
36	0	0	-0.9723	-42.3058	0
37	0	0.0052	-3.5062	-45.4026	1
38	0	0.0084	-2.1935	-50.4456	1
39	0	0	-2.1185	-42.6227	0

40	0	0.015	-2.6923	-49.133	1
41	0	0.9904	-4.0205	-43.4843	3
42	1.7813	0.028	8.9078	-58.9343	1
43	0	0	-3.5162	-34.3235	0
44	0	0	-4.146	-36.4154	0
45	0	0	-2.6802	-34.5214	0
46	0	0	-4.0999	-36.3648	0
47	0	0.0005	-4.053	-33.0628	2
48	0	0	-2.2123	-39.1164	0
49	0	0	-4.1075	-33.1122	0
50	0	0	-1.7961	-39.1621	0
51	0	0	-3.4414	-29.1303	0
52	0	0.0019	-2.1997	-34.6724	1
53	0	0.0016	-3.0278	-30.4643	1
54	0	0.0108	-2.7451	-37.1465	2
55	0	0	3.0391	-72.2022	1
56	0	0	0	-61.7662	0
57	0	0	0	-61.1466	0
58	0	0	-0.74033	-59.7962	0
59	0	0	-1.35915	-58.5762	0
60	0	0	-0.9865	-57.2076	0
61	0	1.0855	3.3839	-58.3369	1
62	0	0	-4.59413	-40.9762	0
63	0	0.0005	-1.2025	-60.3498	2
64	0	0	0	-39.5214	0
65	0	0	-0.5568	-40.2497	0
66	0	0	-0.5162	-49.7484	1
67	0	0	-1.783	-35.1129	0
68	0	0.0812	-3.8245	-33.5117	2
69	0	0.0011	-3.823	-41.0994	1

Table S2. Measured log*K* values and scores calculated by ChemPLP for the training set of guests (see Fig. 2, main text).

Guest	*log*K_{expt}	*ChemPLP Score*	*Guest*	*log*K_{expt}	*ChemPLP Score*	*Guest*	*log*K_{expt}	*ChemPLP Score*
1	3.49	49.81	*19*	4.28	45.54	*37*	4.90	53.86
2	1.15	39.49	*20*	3.94	50.66	*38*	4.30	57.79
3	3.83	48.47	*21*	1.15	47.52	*39*	4.00	49.12
4	1.87	38.88	*22*	1.73	38.51	*40*	1.95	45.94
5	3.96	47.67	*23*	2.11	39.26	*41*	2.08	53.28
6	1.52	35.61	*24*	2.62	40.55	*42*	-1.00	54.68
7	3.88	47.96	*25*	3.32	44.58	*43*	3.70	43.31
8	5.00	53.35	*26*	3.60	46.56	*44*	3.60	44.45
9	1.86	40.64	*27*	4.04	46.04	*45*	3.60	42.13
10	3.49	38.32	*28*	5.15	53.94	*46*	3.48	45.25
11	2.83	43.45	*29*	6.08	59.72	*47*	1.78	49.63
12	2.18	40.87	*30*	5.18	62.76	*48*	3.60	46.59
13	1.48	41.77	*31*	-1.00	62.48	*49*	2.41	46.87
14	4.30	57.43	*32*	-1.00	64.03	*50*	2.30	44.66
15	4.20	54.43	*33*	-1.00	58.15	*51*	0.48	40.70
16	4.88	52.62	*34*	-1.00	47.30	*52*	0.70	42.40
17	3.98	50.59	*35*	-1.00	45.61	*53*	0.90	39.14
18	5.26	46.88	*36*	4.30	49.32	*54*	0.70	46.49

Table S3. Measured log*K* values, and log*K* values calculated by eq. 2 for the training set of guests (see Fig. 3, main text).

Guest	$logK_{expt}$	$logK_{calc}$	*Guest*	$logK_{expt}$	$logK_{calc}$	*Guest*	$logK_{expt}$	$logK_{calc}$
1	3.49	3.28	*19*	4.28	2.92	*37*	4.90	3.27
2	1.15	1.87	*20*	3.94	2.43	*38*	4.30	3.83
3	3.83	3.08	*21*	1.15	1.99	*39*	4.00	3.23
4	1.87	2.34	*22*	1.73	2.35	*40*	1.95	3.65
5	3.96	2.99	*23*	2.11	2.37	*41*	2.08	0.38
6	1.52	1.91	*24*	2.62	2.54	*42*	-1.00	-1.00
7	3.88	3.06	*25*	3.32	2.81	*43*	3.70	2.38
8	5.00	3.45	*26*	3.60	2.92	*44*	3.60	2.47
9	1.86	2.29	*27*	4.04	2.92	*45*	3.60	2.50
10	3.49	2.11	*28*	5.15	3.60	*46*	3.48	2.47
11	2.83	1.86	*29*	6.08	4.24	*47*	1.78	2.21
12	2.18	2.20	*30*	5.18	4.76	*48*	3.60	2.93
13	1.48	1.30	*31*	-1.00	3.69	*49*	2.41	2.21
14	4.30	2.52	*32*	-1.00	4.43	*50*	2.30	2.98
15	4.20	3.09	*33*	-1.00	2.05	*51*	0.48	1.96
16	4.88	3.63	*34*	-1.00	2.94	*52*	0.70	2.56
17	3.98	3.25	*35*	-1.00	2.67	*53*	0.90	2.12
18	5.26	3.16	*36*	4.30	3.34	*54*	0.70	2.67

Table S4. Measured log*K* values, and log*K* values calculated by eq. 3 for the training set of guests (see Fig. 4, main text).

Guest	$logK_{expt}$	$logK_{calc}$	Guest	$logK_{expt}$	$logK_{calc}$	Guest	$logK_{expt}$	$logK_{calc}$
1	3.49	4.02	*19*	4.28	3.58	*37*	4.90	3.02
2	1.15	2.20	*20*	3.94	3.52	*38*	4.30	3.82
3	3.83	3.75	*21*	1.15	2.40	*39*	4.00	3.95
4	1.87	2.83	*22*	1.73	2.85	*40*	1.95	3.58
5	3.96	3.62	*23*	2.11	2.87	*41*	2.08	1.75
6	1.52	2.24	*24*	2.62	3.10	*42*	-1.00	-1.00
7	3.88	3.72	*25*	3.32	3.43	*43*	3.70	2.81
8	5.00	4.20	*26*	3.60	3.55	*44*	3.60	2.89
9	1.86	2.70	*27*	4.04	3.56	*45*	3.60	3.00
10	3.49	2.50	*28*	5.15	4.42	*46*	3.48	2.90
11	2.83	2.12	*29*	6.08	5.24	*47*	1.78	0.71
12	2.18	2.59	*30*	5.18	5.93	*48*	3.60	3.57
13	1.48	1.39	*31*	-1.00	-1.06	*49*	2.41	2.56
14	4.30	4.95	*32*	-1.00	-0.83	*50*	2.30	3.66
15	4.20	3.08	*33*	-1.00	-0.51	*51*	0.48	2.29
16	4.88	4.50	*34*	-1.00	-1.83	*52*	0.70	2.19
17	3.98	3.97	*35*	-1.00	-0.10	*53*	0.90	1.58
18	5.26	3.88	*36*	4.30	4.15	*54*	0.70	1.41

Table S5. Measured log*K* values, and log*K* values calculated using the final scoring function in eq. 3, for an additional set of 15 guests identified by a screen of an in-house library of 3000 compounds (see Fig. 6, main text).

Guest	$logK_{expt}$	$logK_{calc}$	Guest	$logK_{expt}$	$logK_{calc}$
55	6.80	7.12	*63*	4.45	4.10
56	8.00	6.35	*64*	4.18	4.06
57	7.26	6.29	*65*	4.20	4.02
58	6.06	6.00	*66*	4.11	4.08
59	6.09	5.74	*67*	3.60	3.24
60	5.73	5.68	*68*	1.11	0.88
61	5.50	6.73	*69*	3.40	2.51
62	2.88	3.27			

References:

1 ChemCell, 2010 Collaborative Drug Discovery, Inc.

2 TorchV10, http://www.cresset-group.com/products/torch/

3 http://www.cresset-group.com/products/xedtools/

4 Open Babel, http://openbabel.org/
